# Scalp high‐frequency activity differentiates neonates with seizures from healthy neonates and indicates postneonatal epilepsy risk

**DOI:** 10.1111/epi.70025

**Published:** 2025-11-21

**Authors:** Panagiota Karatza, Dorottya Cserpan, Santo Pietro Lo Biundo, Andrea Rüegger, Francesco Pisani, Johannes Sarnthein, Georgia Ramantani

**Affiliations:** ^1^ Department of Neuropediatrics, University Children's Hospital University of Zurich Zurich Switzerland; ^2^ Child Neurology and Psychiatry Unit, Department of Human Neurosciences Sapienza University of Rome Rome Italy; ^3^ Department of Neurosurgery, University Hospital Zurich University of Zurich Zurich Switzerland; ^4^ Zurich Neuroscience Center, Eidgenössische Technische Hochschule University of Zurich Zurich Switzerland

**Keywords:** biomarker, high‐frequency activity, high‐frequency oscillations, neonatal seizures, postneonatal epilepsy risk

## Abstract

**Objective:**

This study investigated whether scalp high‐frequency activity (HFA) rates in neonates with seizures predict postneonatal epilepsy (PNE). It also assessed whether HFA rates differentiate neonates with seizures from healthy neonates and whether they vary by seizure etiology, therapeutic hypothermia, and electroencephalographic (EEG) background activity.

**Methods:**

We included 47 neonates with EEG‐confirmed seizures (nine with neonatal mortality, three lost to follow‐up, 35 with 1‐year follow‐up), and eight healthy neonates. Scalp HFA rates during sleep were determined using an automated detector.

**Results:**

Neonatal seizure etiologies included hypoxic–ischemic encephalopathy (HIE, *n* = 16), structural vascular lesions (SVL, *n* = 14), and neonatal onset genetic epilepsies (*n* = 14). Scalp HFA rates were significantly higher in neonates with seizures (.16 ± .15 HFA/min/channel [ch]) than in healthy neonates (.03 ± .02 HFA/min/ch), with a threshold of .06 HFA/min/ch best differentiating these groups. Among neonates with seizures, those with genetic etiologies had significantly higher HFA rates (.24 ± .19 HFA/min/ch) than those with SVL (.07 ± .05 HFA/min/ch). HFA rates were not associated with EEG background activity and were unaffected by therapeutic hypothermia in neonates with HIE. Of the 35 surviving neonates with seizures, 11 developed PNE, whereas 16 had normal development at follow‐up. Neonates who developed PNE had significantly higher HFA rates (.27 ± .18 HFA/min/ch) than those with normal development (.11 ± .09 HFA/min/ch), with a threshold of .12 HFA/min/ch best differentiating these groups.

**Significance:**

Scalp HFA differentiates neonates with seizures from healthy neonates and may help identify those at higher risk for PNE. These findings support the potential use of scalp HFA as a potential biomarker for seizure monitoring and epilepsy risk stratification in neonates.

**Key points**

- Scalp HFA distinguishes neonates with seizures from healthy neonates.
- HFA rates vary by seizure etiology, with higher rates in genetic epilepsies.
- Therapeutic hypothermia in HIE does not affect HFA rates.
- HFA rates do not correlate with EEG background activity.
- Higher HFA rates are associated with increased risk of postneonatal epilepsy.

## 1 INTRODUCTION

Neonatal seizures are the most prevalent neurological disorder in early life, occurring more frequently during this period than at any other time. These seizures often indicate severe central nervous system (CNS) dysfunction, carrying risks of neonatal death and adverse long‐term outcomes such as developmental delay, neurological deficits, and postneonatal epilepsy (PNE).^1^, ^2^, ^3^ Although most neonatal seizures are acute symptomatic events resulting from perinatal brain injury, 15%–25% represent the first manifestation of epilepsy, often associated with cortical malformations, genetic mutations, or metabolic disorders.^4^, ^5^ Early detection is critical for timely intervention.^6^ Conversely, in up to 85% of neonates with seizures, these seizures are self‐limiting, and these neonates may not develop epilepsy. Thus, distinguishing self‐limiting seizures from neonatal onset epilepsy is essential for patient management and family counseling.^7^, ^8^

Scalp electroencephalography (EEG) is the primary tool for detecting neonatal seizures, as many show no observable clinical symptoms. Continuous EEG monitoring is recommended to facilitate early seizure detection and treatment,^9^ with the goal of reducing seizure burden, as a higher burden is associated with poorer treatment response.^10^ However, continuous EEG is resource‐intensive, costly, and not widely available, even in high‐income settings. In addition, interictal EEG abnormalities—particularly background activity patterns rather than interictal epileptic discharges^2^—may better predict outcomes^11^, ^12^, ^13^, ^14^ than seizure type or etiology,^11^, ^12^, ^15^, ^16^ but definitive diagnostic markers have yet to be established.^4^ Predicting each neonate's seizure and epilepsy risk from an initial standard EEG could optimize resource allocation and reduce unnecessary procedures.

Over the past 2 decades, high‐frequency activity (HFA; >80 Hz) on intracranial^17^ and scalp EEG^18^, ^19^, ^20^, ^21^, ^22^ has received significant attention in epilepsy research. Within this spectrum, high‐frequency oscillations (HFOs) in scalp EEG, originally used to localize seizure‐generating brain regions,^23^ have emerged as potential noninvasive biomarkers for assessing disease severity^24^, ^25^, ^26^ and treatment response.^19^, ^27^, ^28^, ^29^, ^30^ Most studies to date focus on pediatric cohorts,^27^, ^28^, ^31^, ^32^ where higher HFO rates—associated with increased sensitivity—have been observed,^32^ with recent research linking HFOs to the processes of ictogenesis and epileptogenesis.^33^ Studies in children show correlations between higher HFO rates and epilepsy development and severity,^25^, ^34^, ^35^ in line with preclinical studies in animal models^36^ linking HFOs with epilepsy risk following traumatic brain injury. Although neonatal seizures offer a unique paradigm for assessing the potential of scalp HFOs as an EEG biomarker in the pediatric population,^23^, ^37^ HFOs have not yet been explored as predictors of epileptogenesis in neonates.

Early studies using manual detection in small neonatal samples indicate that neonates with seizures can produce epileptic HFOs,^38^, ^39^ with higher HFO rates associated with severely abnormal EEG background activity.^39^ A study using automated detection^37^ found higher HFO rates in neonates with seizures compared to healthy controls, although limited sample sizes limit the generalizability of these findings. It thus remains unclear whether HFOs can reliably differentiate between seizure‐prone and healthy neonatal brains. Most importantly, the previous studies have not investigated whether neonatal HFOs could serve as predictors of long‐term outcomes such as PNE.

Given the complexities of neonatal EEG, we use the broader term HFA throughout, referring to events that may include HFOs as well as other high‐frequency phenomena; we retain “HFO” when citing prior work that used that term. As our primary aim, we investigate whether HFA rates in neonates with seizures can predict the development of PNE. As our secondary aim, we validate that HFA rates are a distinguishing feature between neonates with seizures and healthy neonates and we assess the effect of seizure etiology, therapeutic hypothermia, and EEG background activity on the HFA rate.

## 2 MATERIALS AND METHODS

### 2.1 Patient selection

We retrospectively identified preterm (<37 weeks gestational age) and term neonates (≤30 days old, corrected gestational age ≤ 44 weeks) from our institutional database who underwent scalp EEG between January 2021 and May 2024 at either the Neonatology and Intensive Care Unit of the University Children's Hospital Zurich or the Neonatology Unit of the University Hospital Zurich. Inclusion criteria were as follows: (1) neonates with EEG‐confirmed seizures or healthy neonates without seizures or any neurologic or systemic disease affecting the CNS, (2) scalp EEG with a sampling frequency > 1 kHz, (3) identifiable EEG sleep epochs, and (4) available informed general consent. The first high‐quality scalp EEG recording with ≥5 min of analyzable sleep data was selected for each neonate. Clinical data included gestational age, seizure etiology, treatment such as antiseizure medication (ASM) and therapeutic hypothermia, and EEG background activity. Seizure etiology was classified according to the current framework^5^ for neonatal seizures and epilepsy syndromes into hypoxic–ischemic encephalopathy (HIE), structural vascular lesions (SVL; including acute ischemic stroke, hemorrhage, and other vascular‐induced ischemia), genetic, and other etiologies. HIE was defined by at least one marker of perinatal depression (Apgar score < 5 at 10 min, need for resuscitation at 10 min, cord blood/base deficit pH of <7.00 or ≥15 mmol·L^−1^ within the first hour of life) and a neurologic condition with at least three moderate or severe features on the Sarnat scale within 1–6 h of birth. Trio exome sequencing was performed for suspected genetic epilepsy, focusing on variants in genes linked to epilepsy syndromes or developmental delay.

Survivor outcomes following neonatal seizures were assessed at a 1‐year follow‐up and categorized into three groups: (1) normal development, (2) developmental delay without PNE, and (3) PNE with developmental delay. PNE was defined as one or more unprovoked seizures occurring after the neonatal period (≥44 weeks corrected gestational age) requiring ASM or meeting diagnostic criteria for epilepsy. Neurodevelopmental outcomes were evaluated at corrected ages of 3, 6, and 12 months, and depending on clinical needs afterward. Assessments were performed by neuropediatricians or developmental pediatricians using clinical impression in 12 cases and standardized tests in 21 cases. The standardized tests included the Bayley Scales of Infant and Toddler Development (BSID‐III; *n* = 7)^40^ and the Griffiths Mental Development Scales (GMDS‐III; *n* = 14).^41^ The BSID composite scores for cognition, language, and motor skills range from 40 to 160 (mean = 100, SD = 15), with scores of ≥85 considered normal and of <85 indicating developmental delay. Developmental quotients from the GMDS were classified as normal for scores of ≥80 and abnormal for scores of <80.

Patient data collection and analysis were approved by the local ethics committee (KEK‐ZH PB‐2021‐01245), and all parents provided informed general consent for the reuse of clinical data for research purposes.

### 2.2 Scalp EEG acquisition and data selection

Scalp video‐EEG was performed using 12 electrodes placed according to the international 10–20 system,^37^, ^42^ adjusted for neonate head circumference, at a sampling rate of 1024 Hz with the Micromed EEG recording system (Mogliano Veneto). Electrode impedances were kept at ≤5 kΩ. Extracerebral leads were used for respiratory, electrocardiographic, and surface electromyographic recordings. According to our institutional protocol, EEG recordings included a full cycle of awake, quiet, and active sleep states.^37^, ^42^ If sleep–wake state transitions were unclear, the EEG was recorded for at least 1 h.

EEG was performed in all cases according to the American Clinical Neurophysiology Society (ACNS)^9^ and due to suspected seizures. In healthy neonates, the suspicious episodes were attributed to benign conditions such as sleep myoclonia, jitteriness, or mild respiratory distress. For neonates with seizures, the study utilized the initial EEG after the first seizure.

Expert neurophysiologists and epileptologists marked sleep stages and seizure patterns following the ACNS criteria.^43^ To minimize contamination by muscle artifacts, only sleep epochs were analyzed. EEG segments containing ictal patterns were excluded, along with the 1 min preceding and following the seizure pattern. Segments with visible artifacts and channels with continuous interference were also excluded.

EEG background activity was categorized^42^, ^44^ as follows: (1) normal; (2) mildly/moderately abnormal (e.g., excess sharp activity, absence or reduced frequency of normal patterns, prolonged low‐voltage periods, slightly decreased overall voltage, asymmetries in voltage or frequencies, or asynchrony for age); and (3) severely abnormal (e.g., isoelectric or invariant low‐voltage activity, burst–suppression patterns, or permanently discontinuous activity). Seizure patterns were defined as sudden, repetitive, evolving stereotyped waveforms with a clear onset, evolution in frequency and morphology, and a distinct resolution of the abnormal discharge.^5^

### 2.3 Automated scalp HFA detection

We report HFA on neonatal scalp EEG and do not assume that each detection is a discrete HFO.

We rereferenced to bipolar channels^23^, ^37^ for optimal resolution and artifact elimination by pairing the channels using all possible combinations of neighboring electrodes,^28^, ^30^, ^31^, ^32^, ^45^ resulting in 24 bipolar channels.^37^ Sleep data were divided into segments of 30 s to 2 min^37^ for analysis.

**FIGURE 1:**
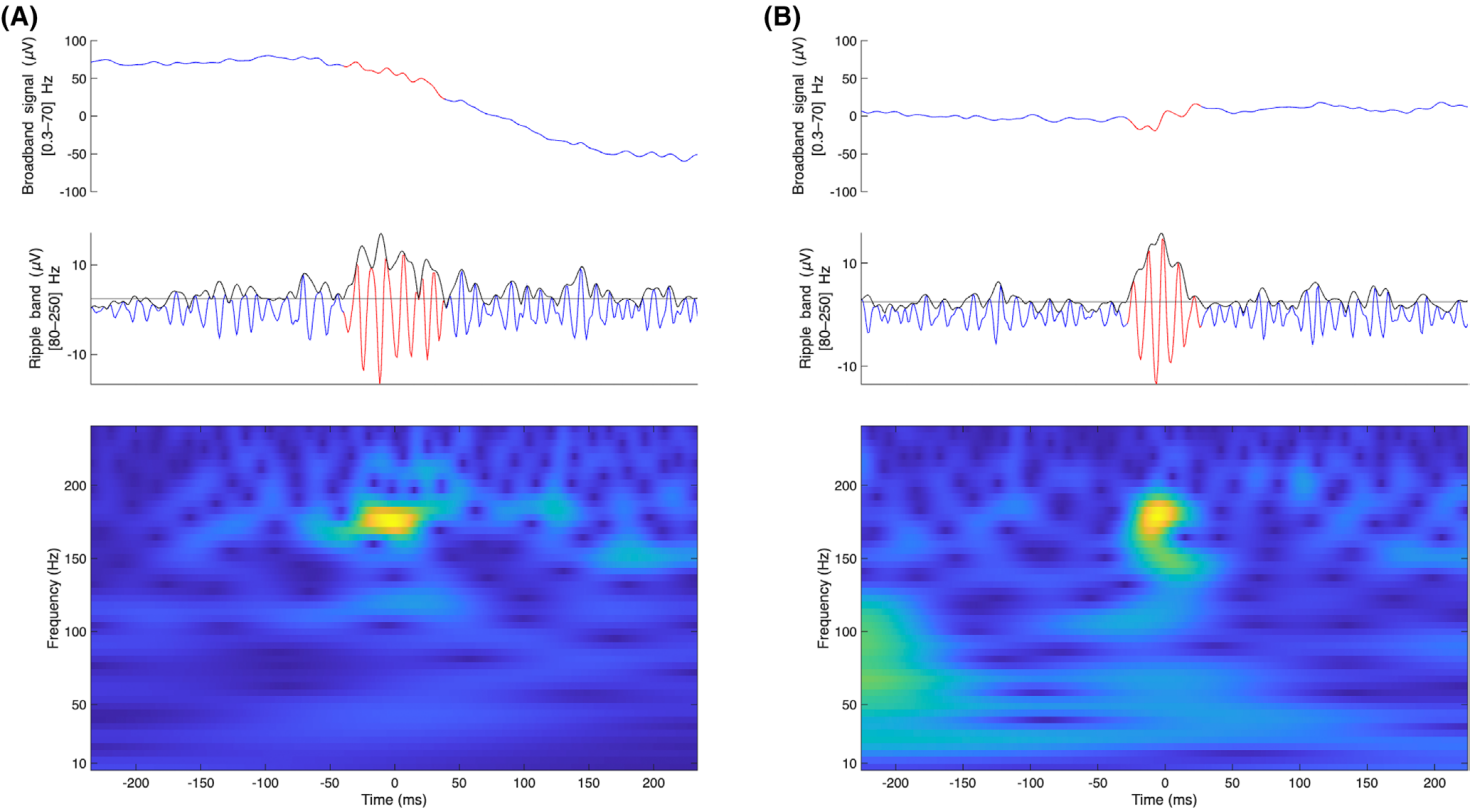
Examples of high‐frequency activity events on the neonatal scalp electroencephalogram. (A) Healthy neonate (signal‐to‐noise ratio [SNR] = 8.6, amplitude = 28.8 μV, seven peaks). (B) Neonate with seizures (SNR = 10.4, amplitude = 28.2 μV, five peaks). Top: Broadband (.3–70 Hz). Middle: 80–250 Hz band‐pass (ripple band). Horizontal black lines indicate baseline amplitude threshold from Stockwell entropy; fluctuating black line indicates Hilbert envelope. Bottom: Time frequency spectrogram. Both examples meet the detection criteria (≥4 peaks above threshold, SNR > 4, prominent high‐frequency peak in the spectrogram).

HFA events were detected using an automated detector^27^, ^46^ previously applied to neonatal data.^37^ The pipeline consisted of three stages.^37^ In Stage I (baseline detection), the EEG signal was filtered with a finite impulse response equiripple band‐pass (80–240 Hz), with stopbands of <70 and >250 Hz, and a 60‐dB attenuation. A baseline amplitude threshold was determined from the Stockwell entropy of data intervals in the ripple band (80–250 Hz). Events of interest (EoIs) were identified when the band‐limited envelope exceeded this threshold for >20 ms and contained ≥4 peaks above threshold. In Stage II (HFA validation), the Stockwell transform was applied to each EoI. Candidate events showing a clear, isolated high‐frequency peak—distinct from surrounding low‐frequency activity—were retained as HFA. In Stage III (artifact rejection), EoIs with peak‐to‐peak amplitude ≥ 40 μV or signal‐to‐noise ratio (SNR) < 4 were discarded. To further adapt the detector for neonatal EEG, we used the FieldTrip *ft_artifact_zvalue* function to compute *z*‐scores across frequency bands. Candidate HFA events were excluded if they overlapped with the following: (1) broadband jump artifacts, identified by *z*‐scores exceeding either five times the interquartile range (IQR) above the median or an absolute value of 15; or (2) muscle artifacts, detected by elevated *z*‐scores in the 110–140‐Hz band. These steps are aimed to reduce false positives^47^ from 1/*f* fluctuations and noncerebral noise. Figure 1 shows two examples of the detected HFA. For each neonate, the average HFA rate was determined by dividing the total number of detected HFA events per minute by the number of analyzed bipolar channels. HFA detection and analysis were performed blinded to clinical features and were not used for clinical decision‐making.

This automated pipeline and thresholds (≥4 peaks, SNR > 4, localized high‐frequency peak) have been used in neonatal EEG previously,^32^ and here we add neonatal‐specific artifact rejection (amplitude ceiling; *z*‐value filters for jump/electromyographic artifacts).

### 2.4 Statistics

We summarized the clinical features of the cohort using descriptive statistics, reporting distributions as mean ± SD. We used permutation tests to compare the means of the HFA rates between neonates with seizures and healthy neonates, between neonates with HIE who underwent therapeutic hypothermia and those who did not, and between neonates who developed PNE and those with normal development. The tests were conducted with 10 000 resamples under a two‐sided alternative hypothesis. Effect size was measured using Hedge *g*‐value. When significant differences were identified, we determined an HFA threshold to differentiate between subgroups of neonates using the maximum Youden index, and calculated specificity, sensitivity, and area under the curve (AUC). For each metric, we determined the 95% confidence intervals (CIs) of proportions (using the Wilson score interval). We analyzed the impact of seizure etiology on HFA rates among the three largest etiology groups (excluding structural brain malformations and metabolic and infectious etiology due to small sample sizes) using Welch analysis of variance (ANOVA), which accounts for unequal variances. Post hoc comparisons were conducted with the Games–Howell post ‐hoc test. The Kruskal–Wallis *H*‐test was applied to explore the relationship between EEG background activity and HFA rates due to the nonnormal distribution of the groups.

## 3 RESULTS

### 3.1 Patient characteristics

**FIGURE 2:**
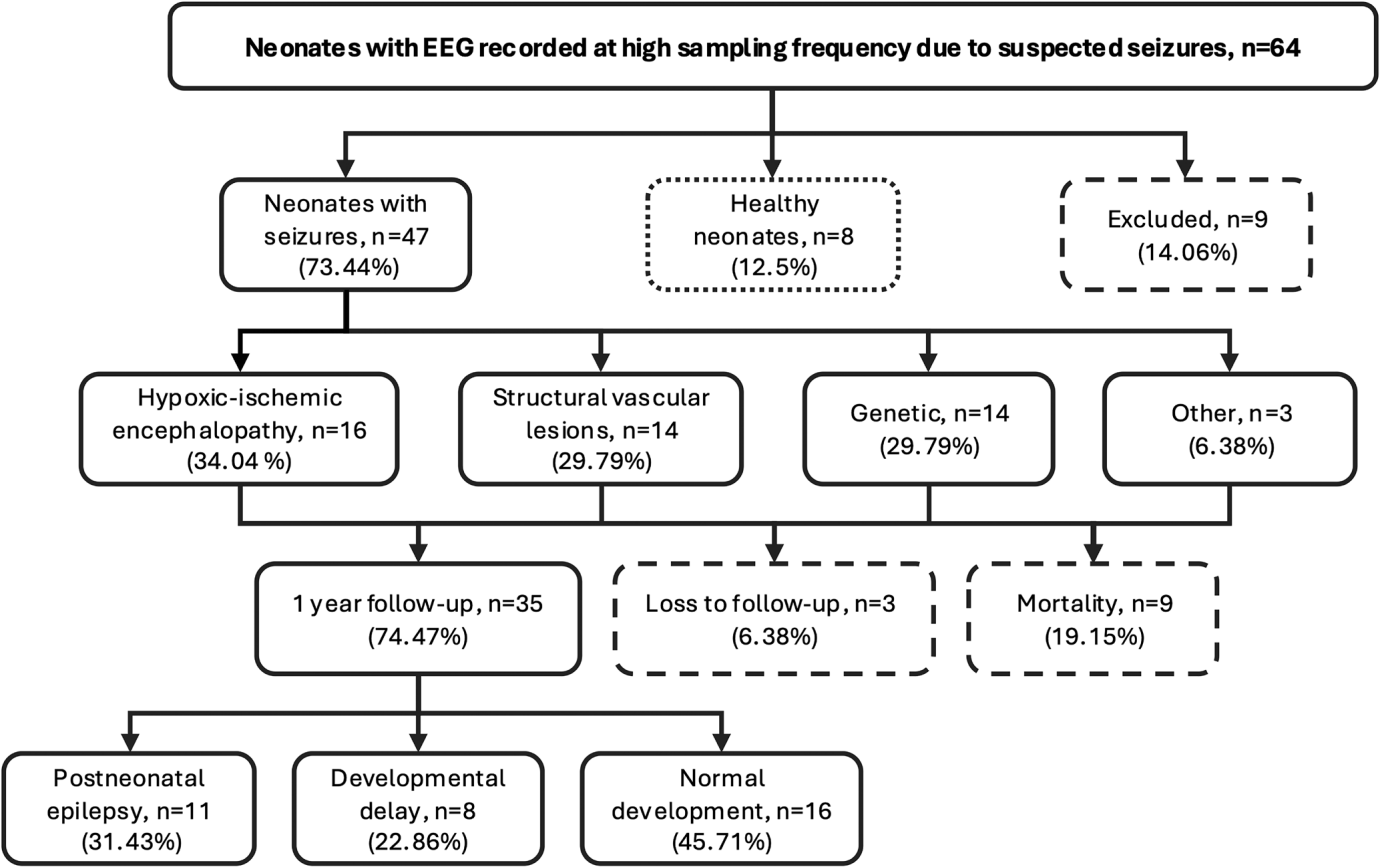
Flowchart of patient selection and outcomes. This flowchart illustrates the selection process for neonates included in the study and summarizes their outcomes at 1‐year follow‐up. It shows the total number of enrolled neonates, excluded neonates, and the final cohort composition, as well as their outcomes, including postneonatal epilepsy, developmental delay, and normal development.

We enrolled 64 neonates (Figure 2) and excluded nine neonates due to EEG recordings with <5 min of sleep (*n* = 6) or poor recording quality (*n* = 3). The final cohort included 47 neonates with EEG‐confirmed seizures (24 female) and eight healthy neonates (2 female) who met the inclusion criteria. Among the neonates with seizures, 41 were full‐term (mean gestational age = 40 ± 1 weeks), and six were preterm (mean gestational age = 33 ± 4 weeks). All healthy neonates were born at term. The mean postnatal age at the time of EEG was 5 ± 5 days for neonates with seizures and 8 ± 8 days for healthy neonates. The total duration of scalp EEG considered for analysis was 2332 min, with an average of 42 ± 26 min per neonate (IQR = 31.93 min).

Seizure etiology included HIE in 16 cases (mild in one case, moderate in seven cases, severe in eight cases^48^), SVL in 14 cases (e.g., acute ischemic stroke, hemorrhage), genetic in 14 cases (e.g., tuberous sclerosis complex and mutations in *PNKP*, *KCNT1*, *POLG1*, *BRAT1*, *SCN2A*), and other causes in three cases. Sixteen neonates presented with status epilepticus. EEG background was classified as normal in 12 cases, mildly to moderately abnormal in 26, and severely abnormal in nine. All 47 neonates with seizures had EEG confirmation at some point during their clinical course; however, only 17 had seizure patterns during the specific EEG segment analyzed in this study. Forty‐four neonates with seizures received ASM shortly before or during the EEG, and eight neonates with HIE underwent therapeutic hypothermia during the EEG. In all healthy neonates, the EEG background was normal, and none received ASM or other CNS medications.

One‐year follow‐up data were available for 35 neonates with seizures; 16 had normal development, eight had developmental delay without PNE, and 11 developed PNE with developmental delay, as shown in Figure 2 and Table 1.

**TABLE 1:** Clinical features of neonates by etiology.

Neonatal cohort	Total, *n* = 55	Neonates with seizures, *n* = 47				Healthy neonates, *n* = 8
		HIE, *n* = 16	SVL, *n* = 14	Genetic, *n* = 14	Other, *n* = 3	
Female	26	7	10	6	1	2
Term	49	13	13	13	2	8
Status epilepticus	16	8	4	3	1	–
Encephalopathy grade						
Severe	8	8	0	0	0	0
Moderate	7	7	0	0	0	0
Mild	1	1	0	0	0	0
Antiseizure medication	44	16	13	13	2	0
Therapeutic hypothermia	8	8	0	0	0	0
Seizure patterns on analyzed EEG	17	7	6	3	1	0
EEG background activity						
Normal	18	6	4	1	1	6
Mildly to moderately abnormal	26	5	10	9	2	0
Severely abnormal	9	5	0	4	0	0
Follow‐up cohort	35	11	10	11	3	–
Normal development	16	6	9	0	1	–
Developmental delay	8	4	1	2	1	–
Postneonatal epilepsy	11	1	0	9	1	–

Abbreviations: EEG, electroencephalogram; HIE, hypoxic–ischemic encephalopathy; SVL, structural vascular lesions.

### 3.2 Scalp HFA rates are higher in neonates with seizures than in healthy neonates

**FIGURE 3:**
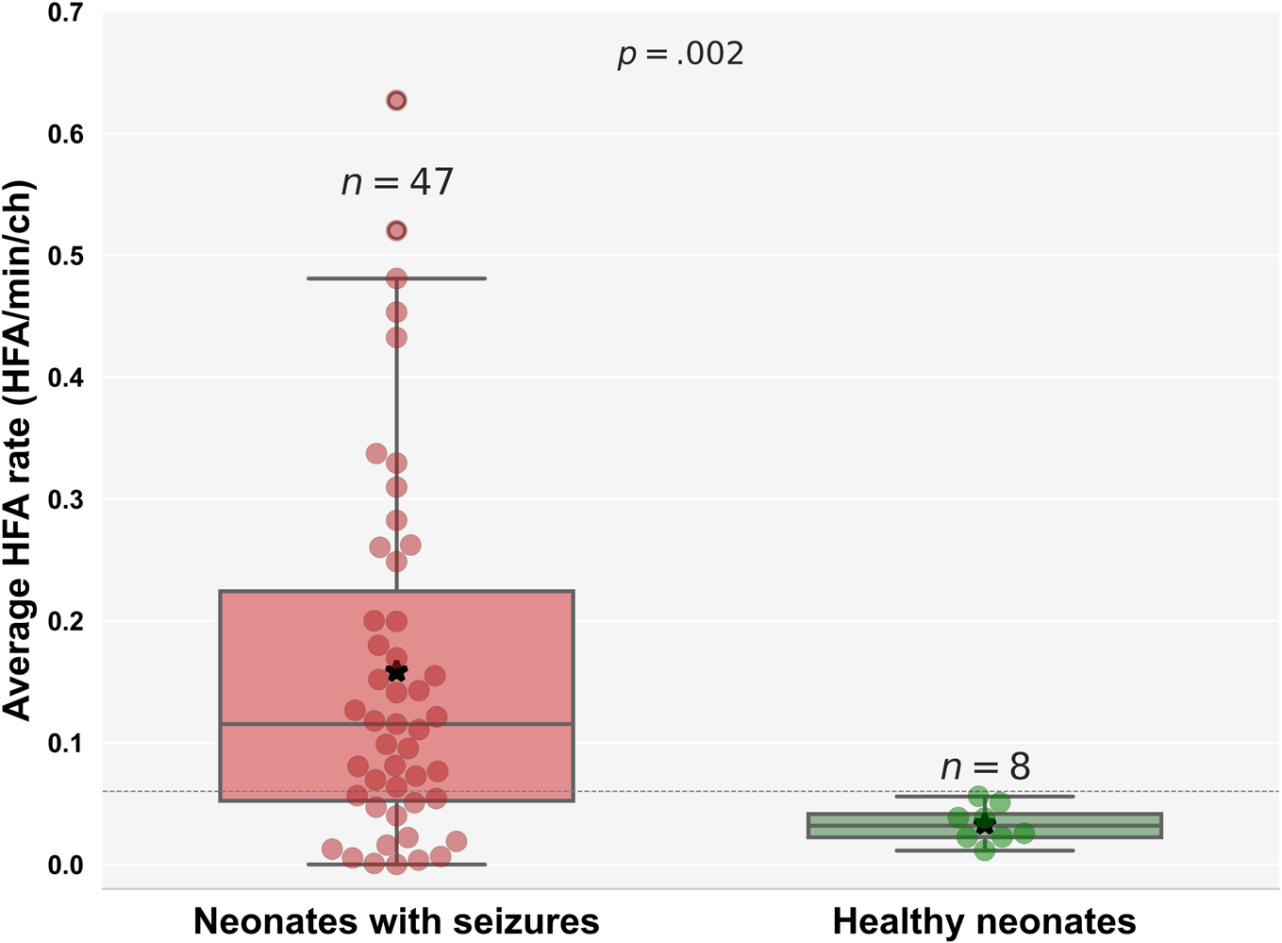
Higher scalp high‐frequency activity (HFA) rates in neonates with seizures compared to healthy neonates. This figure demonstrates that neonates with seizures (*n* = 47, .16 ± .15 HFA/min/channel [ch]) have significantly higher scalp HFA rates compared to healthy neonates (*n* = 8, .03 ± .02 HFA/min/ch; permutation test, *p* = .002). Each red circle represents the average HFA rate for a neonate with seizures, and each green circle represents a healthy neonate. The gray dashed line indicates the optimal HFA threshold (.06 HFA/min/ch) identified by the Youden index to differentiate between neonates with seizures and healthy neonates. Asterisks denote mean HFA values for each group.

The average HFA rates were significantly higher in neonates with seizures (*n* = 47, .16 ± .15 HFA/min/channel [ch]) compared to healthy neonates (*n* = 8, .03 ± .02 HFA/min/ch; permutation test, *p* = .002; Figure 3, Figure S1). Hedge *g*‐value was .87, indicating a large effect size. Using the Youden index, a threshold of .06 HFA/min/ch was identified to best distinguish neonates with seizures from healthy neonates, yielding a specificity of 100% (95% CI = 68%–100%), sensitivity of 72% (95% CI = 58%–83%), and AUC of 80% (95% CI = 67%–90%). The larger variance in HFA rates among neonates with seizures likely reflects the clinical heterogeneity of this population. In particular, very sick neonates—often treated with multiple ASMs and facing higher mortality—tended to show lower HFA rates, likely due to both treatment effects^30^ and their compromised neurological state. In contrast, healthy neonates consistently exhibited low HFA rates.

### 3.3 Scalp HFA rates vary by seizure etiology but not by EEG background activity or therapeutic hypothermia

**FIGURE 4:**
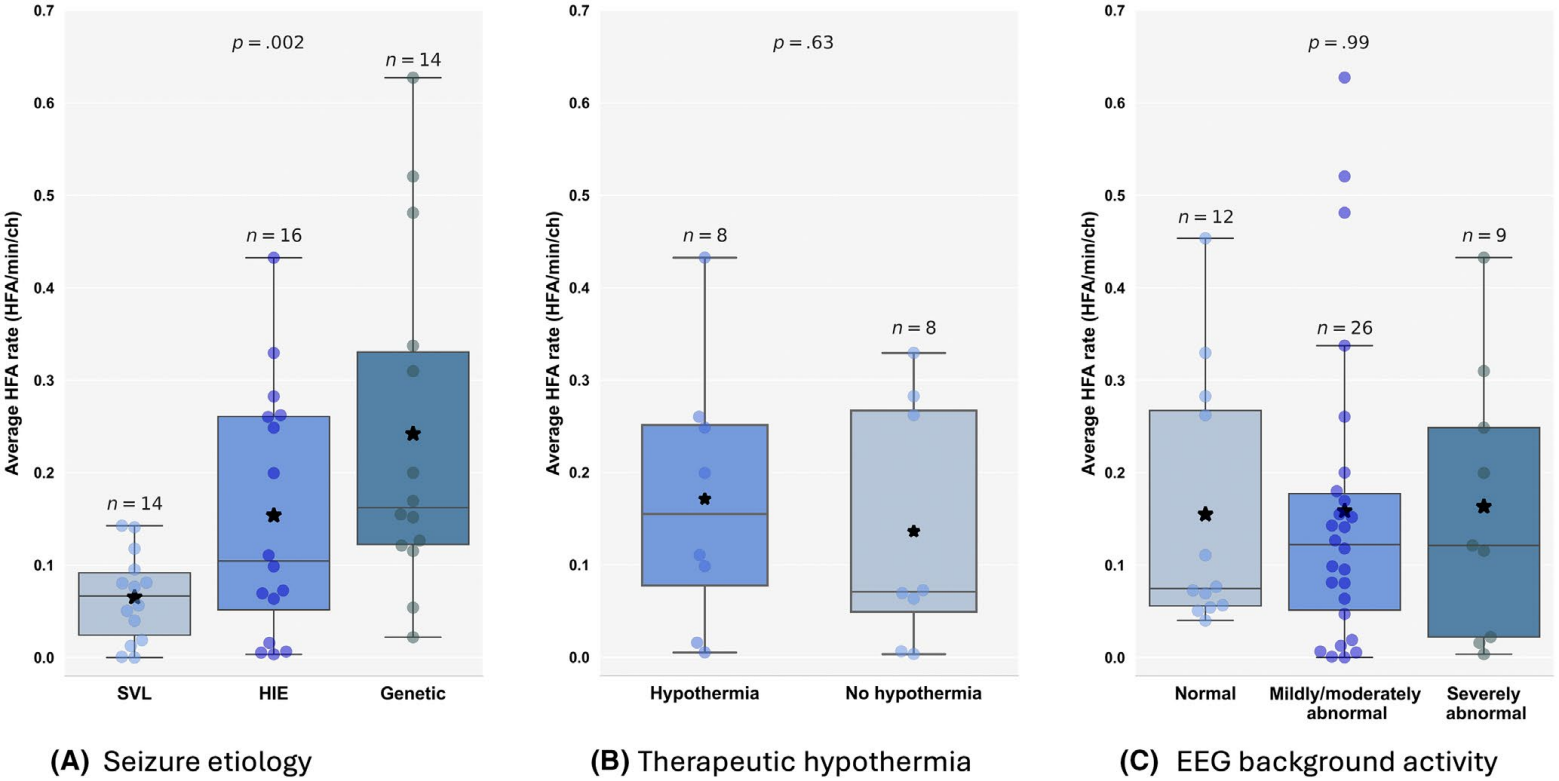
Scalp high‐frequency activity (HFA) rates by seizure etiology, therapeutic hypothermia, and electroencephalographic (EEG) background activity. (A) HFA rates vary significantly by seizure etiology (Welch analysis of variance, *p* = .002), with higher rates in neonates with genetic etiologies (*n* = 14, .24 ± .19 HFA/min/channel [ch]) compared to those with structural vascular lesions (*n* = 14, .07 ± .05 HFA/min/ch; Games–Howell post hoc test, *p* = .004). No significant difference was observed between neonates with genetic etiologies and those with hypoxic–ischemic encephalopathy (*n* = 16, .15 ± .14 HFA/min/ch; Games–Howell post hoc test, *p* = .2). (B) Neonates with hypoxic–ischemic encephalopathy (HIE) who underwent therapeutic hypothermia (*n* = 8, .17 ± .14 HFA/min/ch) did not significantly differ from those with HIE who did not undergo therapeutic hypothermia (*n* = 8, .14 ± .13 HFA/min/ch; permutation test, *p* = .63). (C) No correlation was found between scalp HFA rates and EEG background activity between patients with normal EEG background (*n* = 12, .15 ± .14 HFA/min/ch), mildly/moderately abnormal EEG background (*n* = 26, .16 ± .15 HFA/min/ch), and severely abnormal EEG background (*n* = 9, .16 ± .14 HFA/min/ch; Kruskal–Wallis *H*‐test, *p* = .99). Each circle represents the average HFA rate for an individual neonate. Asterisks indicate the mean HFA rates for each subgroup. SVL, structural vascular lesions.

HFA rates differed significantly based on seizure etiology (Welch ANOVA, *p* = .002; Figure 4A). Neonates with genetic etiologies had significantly higher HFA rates (*n* = 14, .24 ± .19 HFA/min/ch) compared to those with SVL (*n* = 14, .07 ± .05 HFA/min/ch; Games–Howell post hoc test, *p* = .004). No significant difference was found between HFA rates in neonates with genetic etiologies and those with HIE (*n* = 16, .15 ± .14 HFA/min/ch; Games–Howell post hoc test, *p* = .2). Neonates with HIE who underwent therapeutic hypothermia (*n* = 8, .17 ± .14 HFA/min/ch) did not significantly differ from those with HIE who did not undergo therapeutic hypothermia (*n* = 8, .14 ± .13 HFA/min/ch; permutation test, *p* = .63; Figure 4B). Additionally, HFA rates did not correlate with EEG background activity between patients with normal EEG background (*n* = 12, .15 ± .14 HFA/min/ch), mildly/moderately abnormal EEG background (*n* = 26, .16 ± .15 HFA/min/ch), and severely abnormal EEG background (*n* = 9, .16 ± .14 HFA/min/ch; Kruskal–Wallis *H*‐test, *p* = .99; Figure 4C). No significant difference was found in HFA rates between neonates with seizures born at term (*n* = 41, .15 ± .14 HFA/min/ch) and those born preterm (*n* = 6, .20 ± .17 HFA/min/ch; permutation test, *p* = .47).

### 3.4 Scalp HFA rates are higher in neonates who develop postneonatal epilepsy

**FIGURE 5:**
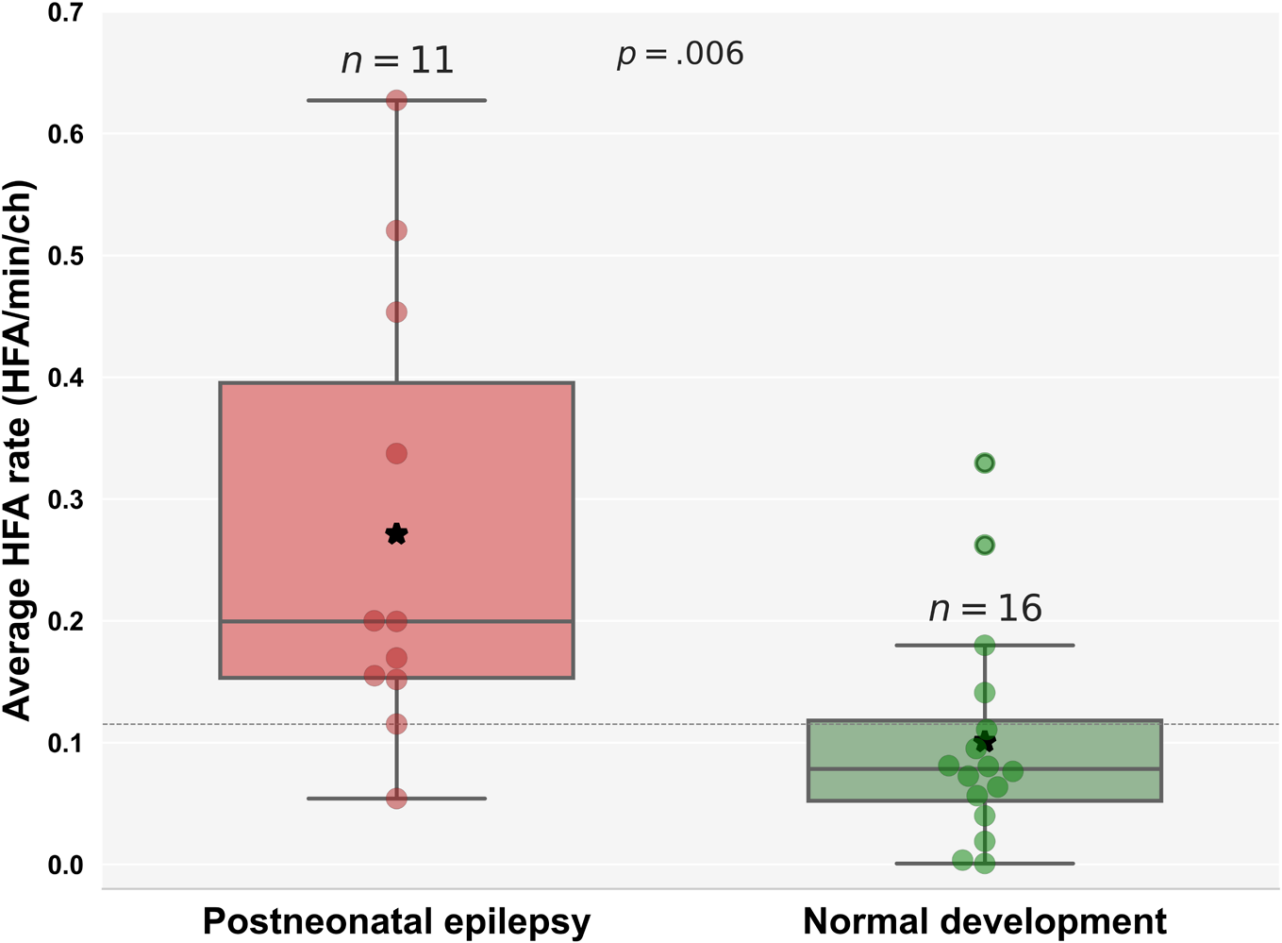
Higher scalp high‐frequency activity (HFA) rates in neonates who later developed postneonatal epilepsy. Neonates who developed postneonatal epilepsy (PNE; *n* = 11, .27 ± .18 HFA/min/channel [ch]) had significantly higher scalp HFA rates compared to those with normal development (*n* = 16, .11 ± .09 HFA/min/ch; permutation test, *p* = .006). Each red circle represents a neonate with PNE, whereas each green circle represents a neonate with normal development. The gray dashed line denotes the optimal threshold (.12 HFA/min/ch) identified by the Youden index for distinguishing neonates at risk of PNE. Asterisks denote the mean values for each group.

At follow‐up, 11 of 35 surviving neonates with seizures developed PNE, whereas 16 had normal development. Those with PNE had significantly higher HFA rates (*n* = 11, .27 ± .18 HFA/min/ch) compared to those with normal development (*n* = 16, .11 ± .09 HFA/min/ch; permutation test, *p* = .006; Figure 5). Hedge *g*‐value was .99, indicating a large effect size. A threshold of .12 HFA/min/ch was identified to differentiate neonates at risk for PNE, with a specificity of 75% (95% CI = 50%–90%), sensitivity of 90% (95% CI = 62%–98%), and AUC of 84% (95% CI = 65%–97%). The neonates who did not survive had relatively low HFA rates (*n* = 9, .1 ± .11 HFA/min/ch), but these did not differ significantly from survivors.

## 4 DISCUSSION

Our findings support scalp HFA as a promising biomarker for assessing seizure and epilepsy risk in neonates, independent of therapeutic hypothermia or EEG background activity. Although further validation in larger independent cohorts is needed, these results highlight the potential of HFA for improving neonatal seizure monitoring and long‐term prognostication.

### 4.1 Higher scalp HFA rates in neonates with postneonatal epilepsy

In our study, neonates with seizures who later developed PNE had significantly higher HFA rates, suggesting that scalp HFA measurement could contribute to epilepsy risk stratification. This finding is relevant for patient management, as it suggests an increased risk of recurrent seizures beyond the neonatal period and emphasizes the importance of early follow‐up. Identifying infants at risk of developing epilepsy following neonatal seizures is essential for improving treatment decisions, avoiding under‐ or overtreatment during key periods of neurocognitive development, and providing informed counseling to families.

Despite extensive research, no reliable clinical predictors currently exist to assess an individual infant's risk of later unprovoked seizures and chronic epilepsy. Because most neonatal seizures are acute symptomatic events and generally self‐limiting, this population is well suited for studying biomarkers that track ictogenesis and epileptogenesis. Although abnormal EEG background activity^2^ has been linked to PNE occurrence^11^, ^12^ and earlier diagnosis,^49^ prior research on neonatal HFOs^37^, ^38^, ^39^ focused exclusively on their role as markers of ictogenesis, without exploring their potential as markers of epileptogenesis.^33^ Our results are in line with pediatric studies showing that scalp HFO rates correlate with seizure risk and disease severity in children with Rolandic spikes^25^, ^36^ and may predict later epilepsy in those with a first unprovoked seizure.^34^ These results extend findings from invasive EEG studies in animal models,^36^ where HFO occurrence—but not rate—following traumatic brain injury was linked to epileptogenesis. By establishing a standardized approach to detecting HFA, our study could help refine prognostic capabilities in neuromonitoring and support a more individualized, precision medicine approach to neonatal seizure management.

In our study, 27.4% of surviving neonates with seizures developed PNE,^16^, ^50^ and all infants with epilepsy also presented developmental delay, highlighting the link between epilepsy and developmental impairment^16^, ^51^ in early brain injury. Ideally, assessing EEG before the first seizure in neonates with suspected brain injury would provide the clearest view of HFA predictive value for PNE and other CNS sequelae, as both seizure activity and ASM can alter EEG patterns and obscure early biomarkers. Beyond neonatal seizures, a reliable, noninvasive biomarker for seizure and epilepsy risk could help identify infants at risk due to trauma, infection, or genetic predisposition, thereby expanding its applicability in neonatal brain health.

### 4.2 Higher scalp HFA rates in neonates with seizures than in healthy neonates

Our study found that neonates with seizures had significantly higher HFA rates than healthy neonates, confirming previous findings,^37^ in a larger cohort and supporting the potential of HFA as a biomarker for identifying seizure risk in neonates. This finding is important for neuromonitoring, because early identification of seizure risk—and thus early intervention—is crucial; treatment efficacy decreases as seizure burden increases,^42^, ^52^ affecting long‐term outcomes.^11^, ^49^

Predicting neonatal seizures remains challenging, as clinical and EEG data alone generally do not reliably predict seizures. Current prediction models rely heavily on manual EEG review, which is limited by small sample sizes and brief review windows.^53^, ^54^ Although certain EEG features, such as background activity, have shown some predictive value,^6^, ^55^, ^56^ reliable independent biomarkers for neonatal seizures are still lacking. Recent machine learning‐based EEG approaches have shown promise for early seizure prediction, but these studies have primarily focused on neonates with HIE.^57^, ^58^ In contrast to previous neonatal HFO studies that relied on manual scoring,^38^, ^39^ our use of an automated detection algorithm^37^ enables scalable analysis, significantly reducing the time and resources needed for manual review. Automated scalp HFA detection could serve as a complementary biomarker, enhancing existing EEG‐based prediction models and extending their applicability beyond HIE to a broader neonatal population. These advances pave the way for future multicenter studies to further validate HFA as a biomarker in larger and more diverse cohorts.

Distinguishing physiological patterns from pathological HFOs remains challenging,^59^, ^60^, ^61^ as physiological patterns in the HFO frequency band can also appear in the scalp EEG of children with epilepsy.^62^ Comparisons of scalp HFO rates between children with and without seizures have largely focused on specific conditions beyond the neonatal period, such as infantile spasms^26^, ^63^ and benign epilepsy with centrotemporal spikes,^35^ with studies consistently showing lower HFO rates in neurologically healthy children, reinforcing the broader utility of HFOs as a biomarker across different age groups.^63^ Given the wide availability of scalp EEG, scalable HFA detection could become a valuable tool for assessing seizure risk in children, starting from the first days of life, and help establish a foundation for broader applications in neuromonitoring and early intervention.

### 4.3 Variation in scalp HFΑ rates by seizure etiology but not by EEG background activity or therapeutic hypothermia

Neonates with genetic seizure etiologies in our study had significantly higher HFΑ rates than those with SVL, suggesting a link between HFΑ rates and specific neonatal seizure etiologies. This novel finding, absent in previous smaller studies,^37^, ^38^, ^39^ is in line with the more severe, refractory course of neonatal onset genetic epilepsies^7^, ^8^ compared to the generally less severe course following perinatal vascular insults.^64^ Notably, in neonates with HIE, epileptogenesis has been linked to higher seizure burden,^49^, ^65^ which may be partially mitigated by therapeutic hypothermia and advances in earlier seizure detection and treatment, potentially contributing to variability in scalp HFΑ rates and outcomes within this group. Our observation contrasts with findings from pediatric studies that reported no differences between cases of focal lesional epilepsy related to focal cortical dysplasia and glioneuronal tumors,^45^ or among epileptic encephalopathy with continuous spike‐and‐wave during sleep related to various etiologies.^29^ This discrepancy may reflect differences in disease severity—and not only etiology—between neonatal onset epilepsies of genetic versus structural vascular origin, as indicated by scalp HFA rates, whereas previous pediatric studies investigated conditions with similar severity across etiologies.

Our study also found no correlation between HFΑ rates and EEG background activity, consistent with our previous findings in a smaller cohort^37^ although contrasting with reports of higher scalp rates in neonates with abnormal background activity.^39^ This suggests that HFΑ rates may more reliably indicate seizure propensity and disease severity than EEG background activity, supporting HFΑ as an independent biomarker.

Therapeutic hypothermia did not impact HFΑ rates in neonates with HIE, despite its known suppressive effect on EEG background activity. Future research should explore other potential influences on HFΑ rates, such as sedatives and ASM, to further validate HFΑ as a robust biomarker in neonatal care.

### 4.4 Future directions

The primary aim of our study was to validate a pattern in the scalp EEG that distinguishes healthy neonates from those who develop epilepsy. HFOs often co‐occur with other epileptiform patterns, which is considered an advantage in both intracranial EEG^66^, ^67^ and scalp EEG.^61^, ^68^ In line with our previous studies,^27^, ^30^, ^31^, ^32^, ^37^, ^45^ we observed no clear differences in HFA/HFO patterns across neonates, infants, children, and adolescents. The automated detection provides consistency, scalability, and objectivity, especially in large datasets. Still, any detection method, automated or expert‐based, will yield some “bycatch” of artifacts or other nonepileptic patterns.

To interpret our findings appropriately, it is important to acknowledge the inherent limitations of the detection approach. Our approach does not allow confirmation of the identity of each detected event. For this reason, we use the term HFA throughout the article, which may include physiological, artifactual, or epileptic components. In this work, HFA rate in the neonatal scalp EEG is an inclusive measure, which besides HFOs might contain high‐frequency components of other sharp activities. The observed association between HFA rate and clinical status does not imply that each detected event reflects pathological activity. The reported performance (AUC = .84) for individual‐level classification of epilepsy development in neonates requires independent validation. The potential clinical relevance of our detection pipeline remains exploratory and requires confirmation in larger, independent studies, ideally with blinded validation.^69^

Our results suggest that the proportion of epileptic HFA may be large enough—and interference from “bycatch” low enough—to allow meaningful group‐level differentiation between neonates with seizures and healthy neonates, and to support the potential use of HFA in epilepsy risk stratification. This supports the potential relevance of our automated detection pipeline.

To better differentiate physiological from pathological HFA, larger longitudinal EEG datasets from healthy neonates of varying gestational ages are needed. However, acquiring such data remains challenging, as healthy neonates are rarely referred for EEG recordings. In our study, the control group is relatively small, reflecting the inherent challenges of recruiting healthy neonates due to ethical and clinical considerations. Nevertheless, the calculated effect size was large, supporting the robustness of our findings and providing a strong basis for further investigation. Further research should assess the impact of commonly used ASMs on scalp HFA rates in neonates, which could help elucidate the mechanisms underlying ASM‐induced changes in cortical excitability. In addition, although EEG segments were carefully selected to minimize seizure‐related influences, the proximity to seizure events remains a consideration. Intracranial studies have demonstrated that HFA can increase within 10–30 s prior to seizure onset.^70^, ^71^ Although this has not yet been systematically demonstrated in scalp EEG studies, in our work, EEG data from 1 min before and after each seizure were excluded to minimize the risk of including preictal activity while preserving a sufficiently large dataset for analysis.

Whereas our study included neonates with mixed seizure etiologies, future work should explore the predictive value of HFA in specific subgroups. Given that genetic epilepsies are strongly associated with PNE, excluding these cases could clarify whether HFA is an independent predictor of PNE in neonates with HIE or SVL. However, as seizure etiology is often unknown at the time of the first EEG, early HFA detection could still aid in risk stratification before a definitive diagnosis is established.

Some subgroup analyses in this study may have been underpowered due to small sample sizes, particularly for etiology, therapeutic hypothermia, and EEG background activity. Post hoc power calculations confirmed that the statistical power for these subgroup comparisons was low, further emphasizing the need for cautious interpretation of these findings. Although key findings support the clinical relevance of HFA, larger etiology‐specific cohorts are needed to determine whether the lack of association in certain subgroups reflects a true absence of effect or limited statistical power. Moreover, as this study was conducted in a single cohort, these preliminary findings require validation in independent datasets to confirm the generalizability of HFA as a biomarker for neonatal seizure and epilepsy risk.

A longer follow‐up period is needed to assess whether HFA predicts both early onset PNE within the first year and also later onset epilepsy. Because many children with PNE develop epilepsy beyond infancy, extended follow‐up could clarify their role in long‐term risk. Nevertheless, the association between HFA and early PNE remains clinically relevant for guiding monitoring and treatment strategies.

The clinical utility of scalp HFA should be further evaluated in prognosis models that integrate multiple data points, including gestational age, clinical features, underlying etiologies, and EEG and magnetic resonance imaging findings. Advances in HFA detection technology,^72^ such as improved noise cancellation and artifact reduction for neonatal intensive care unit settings, could enhance long‐term monitoring with real‐time or even remote detection.^73^ Establishing scalp HFA as a standard tool for assessing at‐risk neonates across a wide range of etiologies and gestational ages may support more targeted epilepsy prevention strategies in early life.

## 5 CONCLUSIONS

These findings highlight the potential of scalp HFΑ as a biomarker for neonatal seizures, PNE risk, and neonatal onset genetic epilepsy. Our study presents a standardized framework for automated scalp HFΑ detection in at‐risk neonates. Further validation in larger cohorts will help determine its role in precision medicine for neonatal seizure management, with the goal of improving prognostication and long‐term outcomes in neonatal care.

## AUTHOR CONTRIBUTIONS

Panagiota Karatza, Dorottya Cserpan, and Georgia Ramantani contributed to the conception and design of the study. Panagiota Karatza, Dorottya Cserpan, Santo Pietro Lo Biundo, Andrea Rüegger, and Georgia Ramantani contributed to the acquisition and analysis of data. Panagiota Karatza, Dorottya Cserpan, and Georgia Ramantani drafted the manuscript and prepared the figures, with significant contributions from Francesco Pisani and Johannes Sarnthein. All authors reviewed, edited, and approved the final version of the manuscript.

## CONFLICT OF INTEREST STATEMENT

None of the authors has any conflict of interest to disclose. We confirm that we have read the Journal's position on issues involved in ethical publication and affirm that this report is consistent with those guidelines.

## Supporting information


Figure S1.

## Data Availability

The data that support the findings of this study are available on request from the corresponding author. The data are not publicly available due to privacy or ethical restrictions.
