# Supplementary material for: Scalp high‐frequency activity differentiates neonates with seizures from healthy neonates and indicates postneonatal epilepsy risk
Source: Epilepsia. 2025 Nov 21;67(3):1303–16. doi: 10.1111/epi.70025 (PMC13007828; doi:10.1111/epi.70025)
Supplement: Supplementary file 1 — Figure S1. [file EPI-67-1303-s001.docx]

**SUPPLEMENTARY MATERIAL**

**A.**

**C.**

**B.**


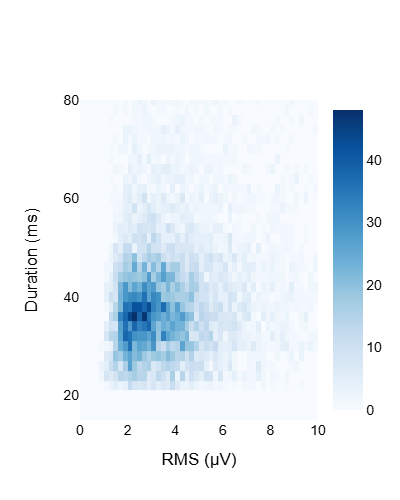

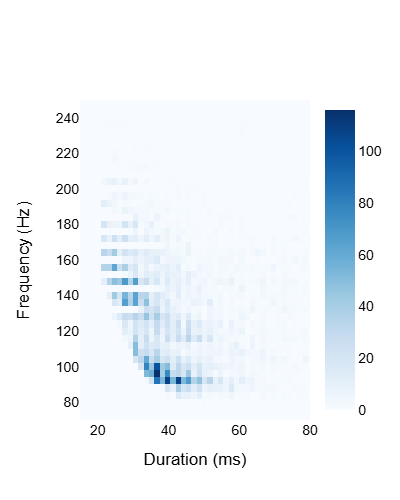

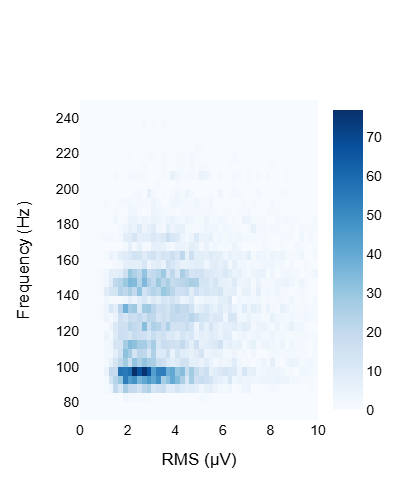


**Supplementary Figure 1.** Characteristics of the HFA events detected in neonates with seizures. The heatmap colors represent the number of events (n = 8,356) with specific values. The mean RMS amplitude was 4.2 ± 2.8 µV, the frequency was 126 ± 28 Hz, and the mean duration was 48.4 ± 29.6 ms. The HFA RMS amplitude was defined as the root mean square of the band-pass filtered signal during the event. Duration was defined as the time during which the Hilbert envelope remained above the detection threshold. Frequency was estimated as the number of peaks minus one, divided by the time (in seconds) between the first and last peak.
